# Impact of Vitamin D Supplementation during Lactation on Vitamin D Status and Body Composition of Mother-Infant Pairs: A MAVID Randomized Controlled Trial

**DOI:** 10.1371/journal.pone.0107708

**Published:** 2014-09-18

**Authors:** Justyna Czech-Kowalska, Julita Latka-Grot, Dorota Bulsiewicz, Maciej Jaworski, Pawel Pludowski, Grazyna Wygledowska, Bogdan Chazan, Beata Pawlus, Anna Zochowska, Maria K. Borszewska-Kornacka, Elzbieta Karczmarewicz, Edyta Czekuc-Kryskiewicz, Anna Dobrzanska

**Affiliations:** 1 Department of Neonatology and Neonatal Intensive Care, The Children’s Memorial Health Institute, Warsaw, Poland; 2 Department of Biochemistry, Radioimmunology, and Experimental Medicine, The Children’s Memorial Health Institute, Warsaw, Poland; 3 Department of Neonatology, Miedzyleski Specialist Hospital, Warsaw, Poland; 4 Department of Neonatology, Holy Family Hospital, Warsaw, Poland; 5 Department of Neonatology, Public Hospital, Otwock, Poland; 6 Department of Neonatology, Warsaw Medical University Hospital, Warsaw, Poland; University of Ottawa, Canada

## Abstract

**Objective:**

The optimal vitamin D intake for nursing women is controversial. Deterioration, at least in bone mass, is reported during lactation. This study evaluated whether vitamin D supplementation during lactation enhances the maternal and infant’s vitamin D status, bone mass and body composition.

**Design and Methods:**

After term delivery, 174 healthy mothers were randomized to receive 1200 IU/d (800 IU/d+400 IU/d from multivitamins) or 400 IU/d (placebo+400 IU/d from multivitamins) of cholecalciferol for 6 months while breastfeeding. All infants received 400 IU/d of cholecalciferol. Serum 25-hydroxyvitamin D [25(OH)D], iPTH, calcium, urinary calcium, and densitometry were performed in mother-offspring pairs after delivery, and at 3 and 6 months later.

**Results:**

A total of 137 (79%) (n = 70; 1200 IU/d, n = 67; 400 IU/d) completed the study. 25(OH)D was similar in both groups at baseline (13.7 ng/ml vs. 16.1 ng/ml; *P = *0.09) and at 3 months (25.7 ng/ml vs. 24.5 ng/ml; *P* = 0.09), but appeared higher in the 1200 IU/d group at 6 months of supplementation (25.6 ng/ml vs. 23.1 ng/ml; *P* = 0.009). The prevalence of 25(OH)D <20 ng/ml was comparable between groups at baseline (71% vs. 64%, *P* = 0.36) but lower in the 1200 IU/d group after 3 months (9% vs. 25%, *P* = 0.009) and 6 months (14% vs. 30%, *P* = 0.03). Maternal and infants’ iPTH, calciuria, bone mass and body composition as well as infants’ 25(OH)D levels were not significantly different between groups during the study. Significant negative correlations were noted between maternal 25(OH)D and fat mass (R = −0.49, *P* = 0.00001), android fat mass (R = −0.53, *P* = 0.00001), and gynoid fat mass (R = −0.43, *P* = 0.00001) after 6 months of supplementation.

**Conclusions:**

Vitamin D supplementation at a dose of 400 IU/d was not sufficient to maintain 25(OH)D >20 ng/ml in nursing women, while 1200 IU/d appeared more effective, but had no effect on breastfed offspring vitamin D status, or changes in the bone mass and the body composition observed in both during breastfeeding.

**Trial Registration:**

ClinicalTrials.gov NCT01506557

## Introduction

Growing evidence suggests that vitamin D deficiency has a negative impact on the skeletal system and may be a potential risk factor for a wide range of diseases, including cancer, cardiovascular disease, hypertension, diabetes, autoimmune diseases, and allergies [1].

Previously, comparable serum 25-hydroxyvitamin D [25(OH)D] levels were reported in lactating and non-lactating women with stable 25(OH)D levels during lactation [2]. On the other hand, decreasing serum 25(OH)D level was observed during lactation in recent studies [3], [4]. Postpartum deterioration of vitamin D status might be explained, at least in part, by less frequent multivitamin intake during lactation compared to pregnancy [5], [6] and decreasing multivitamin intake in the course of lactation [2]. Vitamin D supplementation in lactating women may ensure appropriate maternal vitamin D status in cases of low vitamin D dietary consumption and restricted synthesis in the skin [7]. Most of the recently published RCT in lactating women have been focused on maternal supplementation as a method of achieving appropriate vitamin D status in both, mothers and their breastfed offspring [8]–[11]. It appeared that daily doses of 4000–6400 IU were needed to effectively increase and then to maintain 25(OH)D levels in the range considered by the authors as optimal, in both the infant and its mother. However, the optimal vitamin D intake as well as optimal 25(OH)D level for lactating women (based on estimated maternal needs) still remains not fully established, or at least not accepted worldwide. The recommended vitamin D intake varies from 400 IU/d to 2000 IU/d [7], [12]–[16], but supplementation using less than 1000 IU/d may be inadequate for maintaining an “optimal” 25(OH)D level [17]. Breastfeeding is associated with increased bone turnover leading to release of calcium from the maternal skeleton that leads to bone mass loss during the first 6 months of lactation, with further recovery after weaning [18]–[21]. However, intervention limited to calcium supplementation only had a minimal effect on bone loss during lactation [21]. Increased bone turnover in lactating women may partly be explained by a decreased estradiol level and rise in prolactin, parathyroid hormone and parathyroid hormone-related protein concentrations during lactation [22], [23]. Nevertheless, the precise mechanism of changes in bone metabolism during lactation is still not completely understood. Further, in addition to the body composition indices (fat and lean mass) vitamin D status seems an independent contributor to bone mineral density in girls and women [24], [25]. A recently published RCT of vitamin D supplementation in breastfed infants showed no effect of different oral dosages of vitamin D (from 400 IU/d up to 1600 IU/d) on infants’ bone mineral content [26].

To the best of our knowledge, bone mass and body composition have not been examined in lactating women and their offspring based on the level of maternal vitamin D intake in randomized control trials. We hypothesized that maternal vitamin D supplementation during lactation at a dose of 1200 IU/d, but not 400 IU/d, is more beneficial for both breastfeeding mothers and breastfed infants, when vitamin D status, bone mass and body composition are controlled for. Higher serum 25(OH)D levels, prevalence of vitamin D sufficiency, fat mass loss as well as a lower prevalence of vitamin D deficiency and decrease of bone mass are expected in lactating women.

## Materials and Methods

### Study design

The study was designed as a prospective, double-blinded, randomized, controlled trial of vitamin D supplementation during lactation. The trial was registered at ClinicalTrials.gov (NCT01506557) two months after the enrolment of the first participant by the principal investigator. There were no deviations from the study protocol between the first participant enrolment and the trial registration date. The protocol for this trial and supporting CONSORT checklist are available as supporting information; see Protocol S1, Protocol S2, and Checklist S1.

### Study cohort and setting

Caucasian lactating mothers were recruited from four hospitals in Warsaw city, Poland (52°N) (Miedzyleski Specialist Hospital, Warsaw Medical University Hospital, Holy Family Hospital, Public Hospital in Otwock) between March 2011 and April 2012. Randomization, allocation, and follow-up (6 months) occurred at The Children’s Memorial Health Institute in Warsaw. The follow –up visits took place between May 2011 and September 2012. Healthy women who delivered at term (gestational age 37–42 weeks) a single neonate with a birth weight appropriate for gestational age and declared breastfeeding for the next 6 months were eligible for the study. The exclusion criteria were: maternal and neonatal endocrine disorders (e.g., diabetes mellitus, thyroid disease, disturbed calcium-phosphorus homeostasis), renal and hepatic insufficiency, anticonvulsant treatment, and congenital malformations of the newborn infant.

### Ethics statement

The study has been conducted according to the principles expressed in the Declaration of Helsinki and approved (46/KBE2009) by the Ethics Committee of The Children’s Memorial Health Institute, Warsaw, Poland. The attached study protocol (Protocol S2) is the original version that was submitted to and approved by the Ethics Committee of The Children’s Memorial Health Institute before the trial began. Written informed consent was obtained from each participant.

### Intervention

Women were instructed to ingest two pills daily: one multivitamin tablet containing 400 IU of vitamin D3 and 200 mg of calcium (Prenatal Classic, Puritans Pride, Holbex, Poland) and one masked capsule containing 800 IU of cholecalciferol (VitaDerol forte, Sequoia, Poland) or placebo. The capsules were identical in appearance and prepared with a solution of medium-chain triglyceride oil, masked and certified by Sequoia (Poland). Multivitamin tablets as well as cholecalciferol capsules were produced according to Good Manufacturing Practice guidelines. Vitamin D intervention started approximately 3 weeks after delivery and continued up to 6 months after delivery. All infants received 400 IU/d of vitamin D3 from “twist-off” capsules throughout the study period. According to Polish recommendations, breastfed infants took an additional 25 µg/d of vitamin K1 (VitaDerol +K, Sequoia, Poland) up to 12 weeks of life, then only vitamin D3 (VitaDerol, Sequoia, Poland). Mothers received supplements at baseline and second visit with written instructions on how to use them and were asked to take all unused products for their next visit. Returned pills were recorded at each follow-up visit to calculate compliance and adherence to the intervention between study visits. Compliance was calculated separately for all study products (multivitamins and cholecalciferol) in mothers and infants at 3 and 6 months after delivery.

### Randomization

Lactating mothers were randomly assigned (1∶1) to 400 IU/d (placebo+400 IU/d from multivitamins) and 1200 IU/d (800 IU/d of cholecalciferol+400 IU/d from multivitamins) group using a computerized random number-generator (block size: 4) and stratified by season of delivery (winter: November–May; summer: June–October). A separate randomization list was generated for each season of delivery. The selected physician (not involved in the study in any other way) was responsible for the allocation procedure and provided the allocated study preparation and multivitamins. All investigators (including the principal), study subjects, health care providers, and laboratory staff remained blinded to the mother’s intervention group throughout the study.

### Data collection

A baseline visit occurred within 3 weeks after delivery (V0). A second visit occurred 3 months after delivery (V3) and the last visit 6 months after delivery (V6). At V0, the maternal socio-demographic and health data, prenatal vitamin intake, and neonatal status at birth were recorded using questionnaires. The infant’s body weight, length, head circumference, and maternal weight and height, which were used to determine the maternal body mass index (BMI; kg/m^2^) and the infant’s Ponderal Index (PI; kg/m^3^), were measured at all three visits.

### Laboratory measurements

Blood samples were collected from the mother-infant pairs at every visit. The baseline neonatal biochemical status was analyzed using umbilical cord blood collected at delivery. Non-fasting spot urine samples to determine the urinary calcium/creatinine ratio (UCa/Cr) (normal range: adults <0.78 mmol/mmol, infants <2.55 mmol/l) were obtained from mothers and infants at only V3 and V6. Serum calcium and UCa/Cr measurements were performed within 6 hours of collection, using a Cobas 6000 analyzer (Roche Diagnostics, Basel, Switzerland). Serum samples for total 25(OH)D and intact parathyroid hormone (iPTH) assays were stored at −80°C until assayed. Total serum 25(OH)D (25-hydroxyvitamin D_2_ and 25-hydroxyvitamin D_3_) level was determined using an immunochemiluminescent method (LIAISON, DiaSorin, Sallugia, Italy) at the laboratory, controlled and certified by the International Vitamin D Proficiency – Testing Program (DEQAS). The intra-assay and inter-assay precision for total serum 25(OH)D were <8% and <11%, respectively, both of which were assessed using a control 25(OH)D concentration of 25.1 ng/ml in our laboratory with infant serum samples. Antibody specificities were 100%, 104%, 40%, 17%, and 0% for 25(OH)D_2_, 25(OH)D_3_, 1,25(OH)_2_D_2_, 1,25(OH)_2_D_3_, and 3-epi-25-OHD_3_, respectively. The cross-reactivity with other vitamin D metabolites and detection limit (4 ng/ml) was provided by the manufacturer (LIAISON, DiaSorin, Sallugia, Italy). Serum 25(OH)D <20 ng/ml defined the vitamin D deficiency state, whereas ≥30 ng/ml reflected vitamin D sufficiency [7]. Serum iPTH was determined by a fully automated electrochemiluminescence system (Elecsys 2010, Roche Diagnostics, Basel, Switzerland). The detection limit was 1.20 pg/ml; the manufacturer’s normal range was 15–65 pg/ml and intra-assay and inter-assay coefficients of variation (CV) ≤2.7% and ≤6.5%, respectively.

### Dual energy X-ray absorptiometry (DXA)

Bone mineral content (BMC), bone mineral density (BMD), total lean body mass (LBM), total fat mass (FM), gynoid FM, and android FM were measured by dual energy X-ray absorptiometry (Lunar Prodigy Advance, GE Healthcare, Madison, United States) at every visit. Measurements were made for the total body (BMC and BMD) and the L2–L4 lumbar spine (lumbar BMC and lumbar BMD) in mothers and the total body and total body less head (less head BMC and less head BMD) in infants. DXA acquisition times were less than 5 minutes for mothers and less than 3 minutes for infants. Optimal infant DXA scans without major artifacts were obtained during spontaneous sleep using special infant software provided by the manufacturer. To ensure the reproducibility of results, the infant’s upper extremities were positioned away from the trunk, both the upper and lower extremities were gently bound using a cotton blanket to avoid movement artifacts, and the head was turned to the side. No sedation was used, only a soft silicon pacifier if acceptable by the baby. DXA scans were analyzed by an experienced Certified Clinical Densitometrist (M.J.) with special attention for region of interest (ROI) placing and for artifacts. Changes in body composition measurements were calculated as percentage differences between measurements obtained at the end of the study (6 months postpartum) and baseline values.

### Dietary vitamin D intake

Infant dietary vitamin D intake was calculated at all visits using a questionnaire that assessed the type of feeding (exclusive breastfeeding, partial breastfeeding), daily volume of infant formula consumed, or complementary food ingested during the study period. The vitamin D concentration per 100 ml of infant formula or complementary food was obtained from the manufacturers. Mean daily dietary vitamin D intake was a product of the mean daily volume consumed during the study period and the vitamin D concentration of the formula. A precise calculation of vitamin D intake from breast milk was not possible. Because vitamin D concentration in human milk varies [27], a concentration of 4 IU/100 ml was arbitrarily chosen for further calculation. For statistical purposes, vitamin D intake from breast milk was estimated to be 20 IU/d (500 ml of breast milk) at baseline and 40 IU/d (1000 ml of breast milk) at follow-up visits. Mothers were questioned about average fish consumption (portions/month) during the last 3 months prior to each visit.

### Sunlight exposure

Maternal UV exposure was assessed at every visit using a questionnaire. The duration of outdoor activity Monday–Friday and during weekends, use of sun-blockers, sunbathing, and holidays in sunny countries during last 3 months prior to each visit were recorded.

### Sample size calculation

To detect a statistically significant difference in serum 25(OH)D level (primary outcome) by 4 ng/ml between the intervention groups after vitamin D supplementation at least 64 mother-infant pairs per group were required at 80% power using a two-sided t-test at α = 0.05. It was based on an assumption that each 400 IU/d of vitamin D may increase 25(OHD) level by 2.8–4.8 ng/ml (mean ∼4 ng/ml) and the standard deviation of 25(OH)D measurements was 8 [28], [29]. Concerning the maternal secondary outcomes, the calculated highest group size was 67 participants per group for total body BMC (difference between groups 150 g, SD = 307; 80% power, α = 0.05, a two-sided t-test), and 12 participants per group for total body BMD (difference between groups 0.06 g, SD = 0.05; 80% power, α = 0.05, a two-sided t-test), and 64 participants per group for serum iPTH (difference between groups 5 pg/ml, SD = 10; 80% power, α = 0.05, a two-sided t-test). To account for dropouts, we considered 75 pairs for each group (n = 150). Due to a higher dropout rate at the beginning of the study, the study population was increased to 174 (87 per intervention group).

### Statistical analysis

The normality of the distribution of analyzed data was tested by the Shapiro-Wilk test. A two-factor repeated-measures ANOVA (with Box-Cox transformation for baseline maternal 25(OH)D level and infants’ 25(OH)D levels at birth, and at 3 and 6 months of age) with time and vitamin D dose (group) as factors was used to analyze maternal and infants’ serum 25(OH)D levels. The primary analysis of serum 25(OH)D levels (the separate models for mothers and infants) was modeled as a function of group, time and the interaction between group and time, while accounting for the repeated measurements across subjects. This was followed by analysis of difference between groups at each time point and within groups during time by the Fisher’s LSD test with respect to repeated measurements and adjustment for multiple comparisons. For other analysis (comparison of an absolute value, increment, % change), we used the unpaired t-test for normally distributed variables, U Mann-Whitney for non- normally distributed variables, and chi -squared test/Fisher’s exact test for categorical variables. Spearman correlations were used to assess the association between variables. Data were analyzed using Statistica PL, version 10.0. The results are presented as medians with the first and third quartile (Q1; Q3) unless otherwise indicated. *P*-values<0.05 were considered significant.

**Figure 1:**
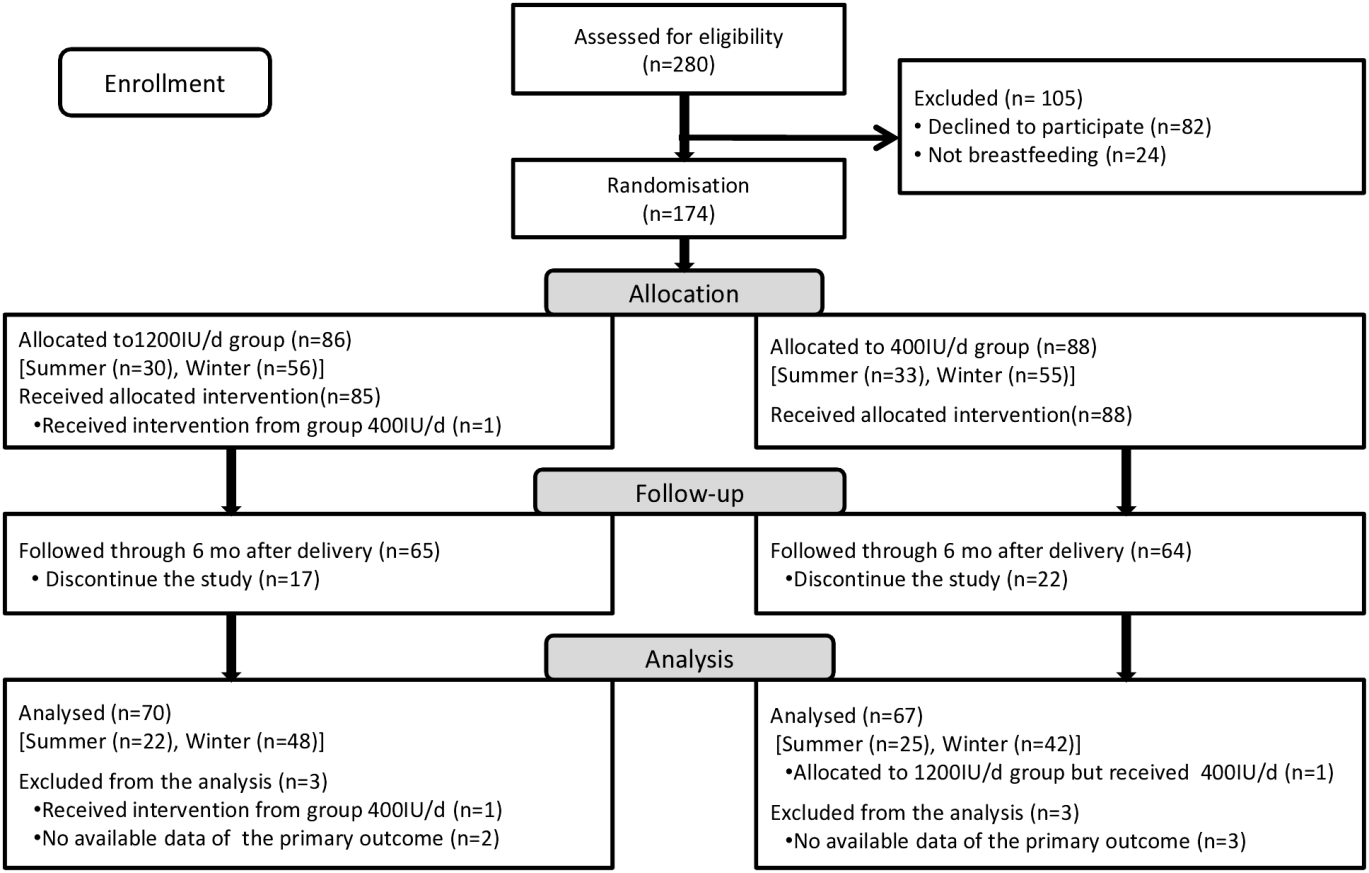
Flow chart of the subjects across the study.

The study was originally planned to conduct an intention-to-treat (ITT) analysis [30]. The ITT approach assesses the effectiveness of improving vitamin D status via different oral vitamin D supplementation, regardless of whether the subject adhered to the dosing regimen. However, under the circumstances only one case of administrative errors occurred (Fig. 1), that patient being analyzed according to the treatment actually received as recommended by Gillings and Koch [30]. Because the primary endpoint was maternal serum 25(OH)D during supplementation, primary analysis was restricted to women who provided a blood sample at baseline and V3 or V6. To support the ITT approach, we decided to impute missing values for V6 using the most recent previous value in 18 (13.1%) participants, as relatively constant biochemical values were noted for V3 and V6. In addition, taking into account adherence, the sub-analysis of primary outcome was performed only in subjects with compliance >80%.

## Results

**Figure 2:**
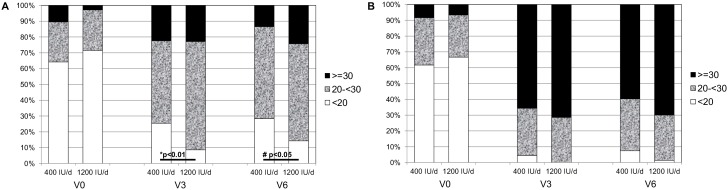
Maternal (A) and infants’ (B) vitamin D status. Percentage of participants with serum 25(OH)D level <20 ng/ml, 20–29.9 ng/ml and >30 ng/ml in both study groups (maternal vitamin D intake 400 IU/d vs. 1200 IU/d). Significant (*P<0.05*) differences between the study groups are shown on the figures.

**Figure 3:**
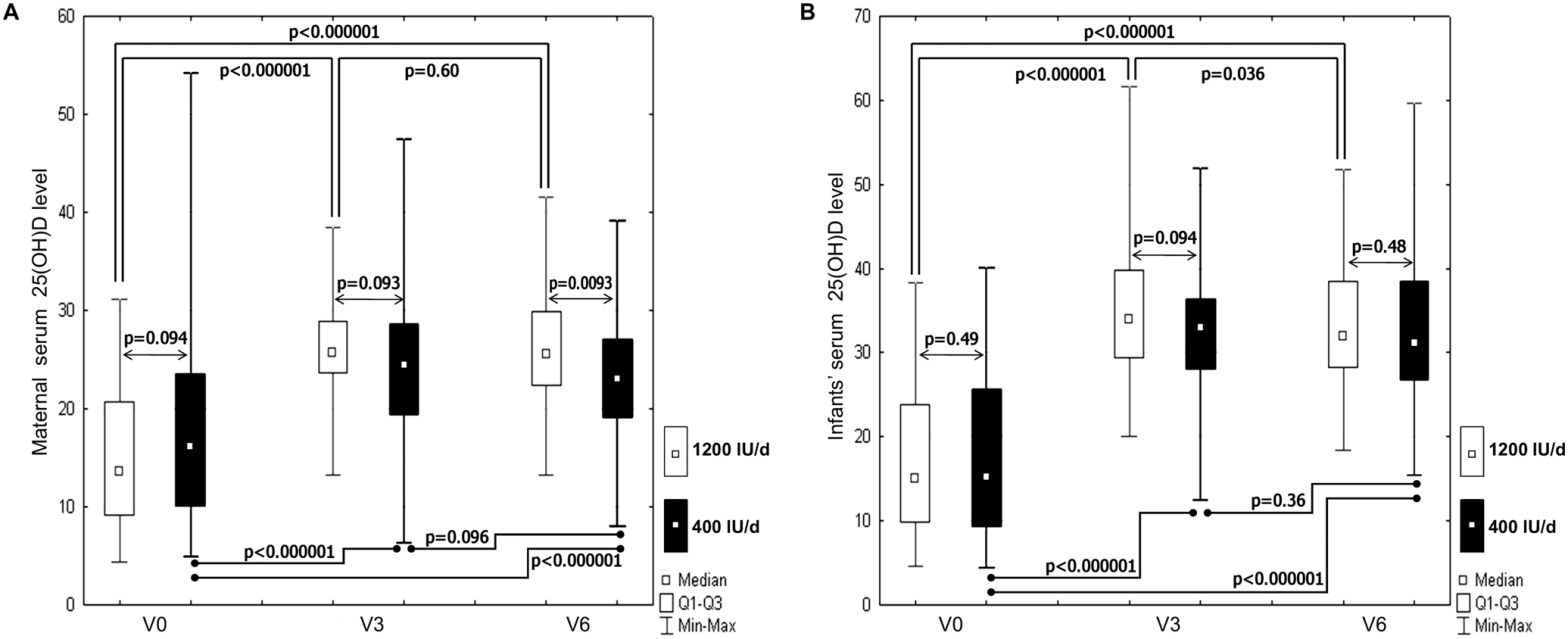
Maternal (A) and infants’ (B) serum 25(OH)D concentrations at baseline and during vitamin D supplementation in the study groups (maternal vitamin D dose: 400 IU/d vs. 1200 IU/d). The effects of group (maternal vitamin D dose) × time interaction, group (maternal vitamin D dose) and time on maternal and infants’ serum 25(OH)D levels were obtained from a two-factor repeated-measures ANOVA. *P*-values for these effects among mothers are 0.00042, <0.000001, 0.25, and among infants 0.12, <0.000001, 0.45, respectively. P-values presented on the figures (A, B) were obtained from post-test analysis (Fisher’s LSD test) for differences in serum 25(OH) concentrations between the study groups and between the study visits. *P*-values<0.05 are significant. The study visits: V0- the baseline visit, V3, V6 – the visits after 3 and 6 months of vitamin D supplementation.

A total of 174 Caucasian lactating mothers were randomly assigned to the treatment group (Fig. 1); 137 (79%) provided data regarding primary outcome [25(OH)D] and were analyzed. The groups were similar in all baseline characteristics, including vitamin D status (Fig. 2, Fig. 3), but a higher maternal education level was found in the 1200 IU/d group (Table 1). The participants who exited the study had similar baseline characteristics as those in the analyzed population with the exception of a 2-fold higher rate of additional formula-feeding (Table 2).

**Table 1:** Sociodemographic and clinical characteristics of the mothers and infants at study enrollment based on vitamin D supplementation group.

Parameter		400 IU/d group	1200 IU/d group	*P*-value between groups
**Mathers**		n = 67	n = 70	
Age, years		30 (27–33)	30 (26–34)	0.89
Baseline BMI, kg/m^2^		24.4 (22.1–27.3)	23.8 (22.1–26.8)	0.49
BMI>30, n (%)		8 (12%)	5 (7%)	0.34
Weight before delivery, kg		77.4 (69–86)	74 (67–83)	0.26
Season at delivery, n (%)	June–October	25 (37%)	22 (31%)	0.47
	November–May	42 (63%)	48 (69%)	
Education, n (%)	>High school	5 (8%)	5 (7%)	0.04
	High school	27 (41%)	15 (21%)	
	College or more	34 (52%)	50 (71%)	
Monthly income, n (%)	<250 Euro/capita	16 (24%)	16 (23%)	0.59
	250–750 Euro/capita	32 (49%)	37 (54%)	
	>750 Euro/capita	18 (27%)	16 (23%)	
Vitamin D during pregnancy,	n (%)	46 (69%)	50 (71%)	0.73
	Daily dose, IU/d	400 (200–800)	400 (200–800)	0.70
	Duration, months	7 (6–9)	7 (5–9)	0.87
**Infants**		n = 67	n = 70	
Age at baseline visit, days		18 (14–22)	15 (13–20)	0.09
Boys, n (%)		29 (44%)	27 (38%)	0.48
Birth weight, g		3500 (3200–3815)	3480 (3280–3640)	0.80
Birth length, cm		55 (53–57)	55 (53–56)	0.25

Data are presented as median and interquartile range (Q1–Q3) or number and (%). *P-value*<0.05 are highlighted.

**Table 2:** Comparison of ITT population with participants lost to follow-up.

Parameter		ITT population	Patients not analyzed	*P*-value
**Mothers**		n = 137	n = 37	
Treatment group, n (%)	1200 IU/d	70 (51%)	16 (43%)	0.33
	400 IU/d	67 (49%)	21 (57%)	
Age, years		30 (27–33)	31 (27–35)	0.56
BMI, kg/m^2^		24.2 (22.1–27.1)	25.3 (22.5–27.4)	0.36
BMI>30, n (%)		13 (10%)	2 (5%)	0.15
Season at delivery, n (%)	June–October	47 (34%)	16 (43%)	0.32
	November–May	90 (66%)	21 (57%)	
Education	>High school	10 (7%)	5 (14%)	0.32
	High school	42 (31%)	13 (36%)	
	College or more	84 (62%)	18 (50%)	
Monthly income, n (%)	<250 Euro/capita	32 (24%)	12 (33%)	0.38
	250–750 Euro/capita	69 (51%)	19 (53%)	
	>750 Euro/capita	34 (25%)	5 (14%)	
Vitamin D intake during pregnancy	Yes, n (%)	96 (70%)	27 (73%)	0.67
	Daily dose, IU/d	400 (200–800)	800 (400–800)	0.14
	Duration, months	7.0 (5.6–9.0)	6.0 (4.6–8.0)	0.17
Outdoor activity, hours	Mon-Fri	2.5 (2.0–4.0)	2.0 (1.9–4.6)	0.96
	Sat-Sun	3.5 (2.0–5.0)	4.0 (2.0–6.0)	0.34
Baseline 25(OH)D, ng/ml		15.2 (9.9–21.5)	15.8 (9.9–26.6)	0.28
25(OH)D >30 ng/ml, n (%)		9 (7%)	3 (8%)	0.72
25(OH)D <20 ng/ml, n (%)		93 (68%)	21 (57%)	0.63
**Infants**		n = 137	n = 37	
Boys, n (%)		56 (41%)	15 (41%)	0.97
Birth weight, g		3480 (3248–3756)	3450 (3180–3756)	0.79
Birth length, cm		55 (53–56)	54 (53–56)	0.22
Head circumference at birth, cm		34 (33–35)	34 (32–35)	0.29
Additional formula feeding, n (%)		21 (15%)	12 (32%)	0.02
Baseline 25(OH)D, ng/ml		15.2 (9.4–24.7) (n = 120)	12.7 (9.0–28.9) (n = 28)	0.84

Data are presented as median and interquartile range (Q1–Q3) or number and (%). *P-value*<0.05 are highlighted.

### Vitamin D status

Maternal 25(OH)D level was similar in both groups at baseline and after 3 months of vitamin D supplementation, however, at V6 significantly higher 25(OH)D levels were noted in the 1200 IU/d group (Fig. 3). The effect of group (vitamin D dose) x time interaction (*P* = 0.0004) and the effect of time (P<0.0001) on maternal serum 25(OH)D level were statistically significant, but the effect of group (vitamin D dose) was not (*P* = 0.25). The mean maternal 25(OH)D increment was 5.5±8.4 ng/ml in the 400 IU/d group and 10.8±9.4 ng/ml in the 1200 IU/d group (*P* = 0.0009) after 6 months of vitamin D supplementation. Baseline maternal 25(OH)D <20 ng/ml was noted in 67.9% of mothers postpartum and 64.2% of cord blood samples. The prevalence of maternal vitamin D deficiency was significantly higher in the 400 IU/d group at V3 (25.4% vs.8.6%; *P* = 0.009) and V6 (29.9% vs. 14.3%; *P* = 0.028) (Fig. 2). On the other hand, the prevalence of vitamin D sufficiency (≥30 ng/ml) was comparable across the intervention groups (400 IU/d vs. 1200 IU/d) during vitamin D supplementation, at V3 (22.4% vs. 22.8%; *P* = 0.95) and V6 (13.4% vs.24.9%; *P* = 0.1) (Fig. 2). As expected, a significant increase in 25(OH)D occurred in breastfed infants supplemented with 400 IU/d of vitamin D, and no differences were found between the study groups (Fig. 3). The effect of group (vitamin D dose) x time interaction (*P* = 0.12) and the effect of group (*P* = 0.45) on infants’ serum 25(OH)D levels was not statistically significant, while the effect of time was statistically significant (*P*<0.0001) (Fig. 3). The prevalence of infants’ vitamin D deficiency and sufficiency was also comparable across the study groups (Fig. 2). However, a higher percentage of vitamin D sufficient infants (up to 70%) than mothers was noted in both study groups (Fig. 2). There was a strong correlation between postpartum maternal and cord blood 25(OH)D levels in the 400 IU/d group (R = 0.86, *P*<0.0001) and in the 1200 IU/d group (R = 0.89, *P*<0.0001), respectively. A correlation between maternal and breastfed infants’ 25(OH)D levels was still significant after 3 and 6 months of vitamin D supplementation but appeared weaker than the postpartum correlation: in the 400 IU/d group at V3 R = 0.35 (*P* = 0.003) and at V6 R = 0.4 (*P* = 0.0009), and in the 1200 IU/d group at V3 R = 0.3 (*P* = 0.01) and at V6 R = 0.39 (*P* = 0.0007), respectively.

Maternal and infant dietary vitamin D intake, sun exposure, and compliance are presented in Table 3. In a sub-analysis that included only participants with compliance >80%, maternal 25(OH)D appeared not significantly different between the study groups: 24.7 ng/ml (19.65–30.32 ng/ml) vs. 25.8 ng/ml (24–30.1 ng/ml) at V3 (*P* = 0.08) and 23.3 ng/ml (18.95–27.95 ng/ml) vs. 25.1 ng/ml (21.73–30.08 ng/ml) at V6 (*P* = 0.14) in the 400 IU/d and 1200 IU/d groups, respectively.

**Table 3:** Vitamin D intake, sun exposure, and compliance.

Parameter	Time	400 IU/d group	1200 IU/d group	*P*-value between groups
**Mothers**		n = 67	n = 70	
Fish consumption, portion/month	Baseline	2 (1–4)	2 (1–4)	0.62
	3 months	2 (1–4)	2 (1–4)	0.36
	6 months	2 (1–4)	2 (1–4)	0.86
Outdoor activity (Mon–Fri), hours	Baseline	2.5 (2.0–4.0)	2.0 (1.5–4.0)	0.21
	3 months	2.0 (1.0–3.0)	2.0 (1.8–3.5)	0.28
	6 months	2.0 (1.3–3.9)	2.0 (1.0–4.0)	0.68
Outdoor activity (Sat–Sun), hours	Baseline	3.5 (2.0–5.4)	3.0 (2.0–4.1)	0.27
	3 months	2.0 (1.3–3.4)	2.5 (1.8–4.5)	0.20
	6 months	3.0 (1.5–4)	2.5 (1.3–3.5)	0.77
Maternal sunblock usage, n (%)	Baseline	18 (27%)	17 (26%)	0.73
	3 months	6 (9%)	10 (14%)	0.34
	6 months	9 (13%)	7 (10%)	0.59
Maternal sunbathing, n (%)	Baseline	13(19%)	6(9%)	0.07
	3 months	8 (12%)	8 (11%)	0.93
	6 months	14 (21%)	14 (20%)	0.60
Holidays in sunny country, n (%)	Baseline	5 (8%)	7 (10%)	0.77
	3 months	0 (0%)	1 (1%)	0.49
	6 months	2 (3%)	3 (4%)	0.79
Mean compliance (Multivitamins), %	3 months	86%	92%	0.002
	6 months	86%	89%	0.03
Mean compliance (Cholecalciferol/placebo), %	3 months	89%	93%	0.02
	6 months	76%	88%	0.08
**Infants**		n = 67	n = 70	
Exclusive breastfeeding, n (%)	Baseline	57 (85%)	59 (84%)	0.90
	3 months	50 (75%)	53 (76%)	0.86
	6 months	2 (3%)	11 (16%)	0.03
Vitamin D intake from diet, IU/d	3 months	40 (40–40)	40 (40–40)	0.50
	6 months	40 (40–235)	40 (40–174)	0.36
Mean compliance, %	3 months	88%	89%	0.31
	6 months	85%	87%	0.73

Data are presented as median and interquartile range (Q1–Q3) or number and (%). *P-value*<0.05 are highlighted.

### PTH levels

Maternal and infants’ iPTH levels at baseline and increment (ΔiPTH) during the study period were comparable across intervention groups at every visit (*P*>0.05). However, different iPTH fluctuations at consecutive visits were observed (Table 4). A significant, negative correlation was noted between maternal iPTH and maternal 25(OH)D throughout the study (V0: R = −0.43, *P* = 0.00001; V3: R = −0.23, *P* = 0.008; V6: R = −0.24; *P = *0.005). A similar weak correlation was found in infants at V3 (R = −0.23, *P = *0.006) and V6 (R = −0.21, *P* = 0.01). However, when cord blood was controlled for, the correlation was not significant (R = 0.001, *P = 0.99*).

**Table 4:** Serum iPTH and calcium concentrations, and urinary Ca/Cr ratio during 6 months of vitamin D supplementation.

Parameter	Time	400 IU/d group	1200 IU/d group	*P*-value between groups
**Mothers**		n = 67	n = 70	
iPTH, pg/ml	Baseline	30.4 (20.2–43.5)	28.6 (20–42)	0.54
ΔiPTH, pg/ml	Baseline to 3 months	−8.7±18.2	−9.0±15.9	0.70
	3 to 6 months	4.1±10.4	3.5±10.3	0.75
Serum Ca, mmol/l	3 months	2.43 (2.36–2.49)	2.44 (2.38–2.52)	0.54
	6 months	2.37 (2.33–2.43)	2.40 (2.36–2.47)	0.13
UCa/Cr ratio, mmol/mmol	3 months	0.22 (0.12–0.38)	0.3 (0.16–0.43)	0.17
	6 months	0.19 (0.08–0.34)	0.23 (0.11–0.41)	0.27
**Infants**		n = 67	n = 70	
iPTH, pg/ml	Baseline	4.8 (3.7–7.1) (n = 61)	4.5 (3.8–5.8) (n = 60)	0.69
ΔiPTH, pg/ml	Baseline to 3 months	10.7±10.0	11.5±6.3	0.80
	3 to 6 months	5.1±8.4	3.0±6.4	0.08
Serum Ca, mmol/l	3 months	2.66 (2.61–2.73)	2.67 (2.62–2.74)	0.76
	6 months	2.62 (2.57–2.68)	2.65 (2.62–2.7)	0.009
UCa/Cr ratio, mmol/mmol	3 month	1.58 (1.02–2.40)	1.51 (0.89–2.47)	0.55
	6 months	1.26 (0.87–1.81)	1.47 (0.86–2.27)	0.17

Data presented as median (interquartile range: Q1–Q3) or mean ± SD. *P-value*<0.05 are highlighted.

Ca- calcium, UCa/Cr ratio- urinary calcium creatinine ratio in spot urine, ΔiPTH- increment of iPTH concentration between the study visits.

### Calcemia and calciuria

No significant differences were found between intervention groups with regard to maternal calcemia and maternal and infants’ calciuria (Table 4). No cases of hypercalcemia were found among participants. Hypercalciuria occurred in 3 mothers from the 400 IU/d group and 1 mother from the 1200 IU/d group at V3, and in 3 mothers from the 400 IU/d group at V6. Serum 25(OH)D concentrations were between 19 ng/ml and 28 ng/ml among the above-mentioned cases. Hypercalciuria was noted in 27 (20.44%) infants at V3 (13 infants of mothers receiving 400 IU/d, and 15 infants of mothers receiving 1200 IU/d; *P* = 0.77). At V6, hypercalciuria was observed in 10.95% of infants (5 from the 400 IU/d group, 10 from the 1200 IU/d group; *P = *0.20).

### Bone mass and body composition

**Figure 4:**
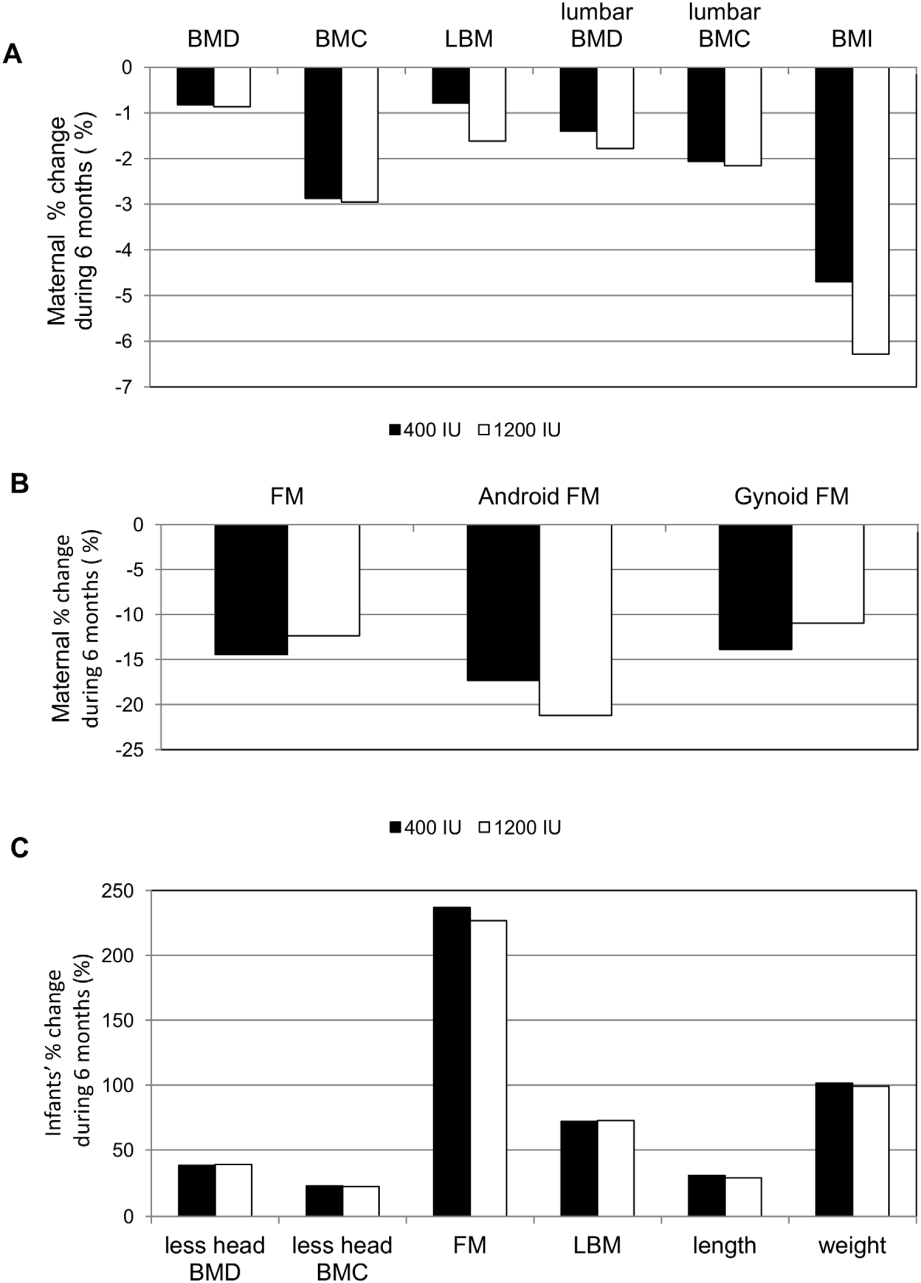
Maternal (A, B) and infants’ (C) % change (between baseline and 6 months visit) in body composition and anthropometric parameters in the study groups during vitamin D3 supplementation. No significant difference between the study groups for all variables (*P*-values*>0.05).* BMD *-* bone mineral density, less head BMD - total body less head mineral density, BMC -bone mineral content, less head BMC - total body less head mineral content, LBM - total lean body mass, BMI - body mass index, FM - total fat, android FM – android fat mass, gynoid FM – gynoid fat mass.

No significant differences were found between the intervention groups with respect to maternal DXA measurements at any time point (Table 5). The mean maternal percent change in body composition parameters during the 6 months of breastfeeding was comparable between the intervention groups (Fig. 4). However, maternal total body fat mass was comparable between groups, significant negative correlations were noted between maternal 25(OH)D level and maternal fat mass (R = −0.49, *P* = 0.00001), android fat mass (R = −0.53, *P* = 0.00001), and gynoid fat mass (R = −0.43, *P* = 0.00001) after 6 months of vitamin D supplementation. The maternal 25(OH)D increment from V0 to V6 inversely correlated only with maternal android fat mass (R = −0.18, *P = *0.048). Significant negative correlations were also noted between maternal 25(OH)D and maternal total body BMC (R = −0.25, *P* = 0.006) and lumbar BMC (R = −0.18, *P* = 0.046), but not total body BMD (R = −0.13, *P* = 0.15) and lumbar BMD (R = −0.14, *P* = 0.13). As expected, an increase in the infants’ less-head total body BMC, BMD, LBM, and FM coincided with increasing infants’ weight and length, with no significant differences between study groups (Fig. 4, Table 6).

**Table 5:** Maternal anthropometry and body composition throughout the study.

Parameter	Time	400 IU/d group	1200 IU/d group	*P*-value between groups
**Mothers**		(n = 67)	(n = 70)	
Weight (kg)	Baseline	66.3 (59.1–77)	65.3 (58–73)	0.35
	3 months	64.5 (58–74)	62.8 (57–70)	0.25
	6 months	63 (55–71)	61.5 (55–68)	0.33
BMI (kg/m^2^)	Baseline	24.4 (22.2–27.3)	23.8 (22.1–26.8)	0.49
	3 months	23.5 (21.4–25.6)	22.8 (21.1–25.7)	0.26
	6 months	23.3 (20.8–25.5)	22.6 (20.1–24.7)	0.59
Total body BMC (g)	Baseline	2545 (2227–2800)	2490 (2201–2741)	0.31
	3 months	2517 (2212–2724) (n = 66)	2456 (2164–2596)	0.29
	6 months	2569 (2161–2764) (n = 59)	2410 (2141–2595) (n = 62)	0.19
Lumbar BMC (g)	Baseline	48.3 (44.1–57.2)	47.5 (43.9–53.8)	0.34
	3 months	47.2 (42.0–55.6) (n = 66)	47.0 (41.6–52.2)	0.46
	6 months	48.8 (42.1–56.3) (n = 59)	47.0 (42.8–53.3) (n = 62)	0.21
Total body BMD (g/cm^2^)	Baseline	1.14 (1.09–1.18)	1.12 (1.07–1.17)	0.23
	3 months	1.13 (1.07–1.18) (n = 66)	1.12 (1.06–1.17)	0.41
	6 months	1.13 (1.08–1.19) (n = 59)	1.11 (1.06–1.16) (n = 62)	0.16
FM (kg)	Baseline	24.25 (19.84–32.57)	23 10 (18.89–29.17)	0.39
	3 months	23.03 (17.39–29.29) (n = 66)	21.47 (17.27–26.29)	0.44
	6 months	21.54 (16.07–27.60) (n = 59)	20.12 (15.11–25.90) (n = 62)	0.46
LBM (kg)	Baseline	39.21 (36.50–42.93)	38.69 (36.64–41.30)	0.43
	3 months	38.81 (35.76–41.84) (n = 66)	37.57 (35.84–40.91)	0.20
	6 months	39.01 (36.79–41.63) (n = 59)	38.39 (35.92–40.49) (n = 62)	0.16

Data presented as median (interquartile range: Q1–Q3). No significant differences between groups, all *P-value>*0.05. (*BMD -* bone mineral density, less head BMD - total body less head mineral density, BMC -bone mineral content, less head BMC - total body less head mineral content, LBM - total lean body mass, BMI - body mass index, FM- total fat, android FM – android fat mass, gynoid FM – gynoid fat mass).

**Table 6:** Infants’ anthropometry and body composition throughout the study.

Parameter	Time	400 IU/d group	1200 IU/d group	*P-*value between groups
		n = 67	n = 70	
Weight (g)	Baseline	3880 (3555–4198)	3715 (3415–4045)	0.12
Δ Weight (g)	Baseline- 3 months	2205 (1870–2650)	2200 (1770–2625)	0.68
	Baseline- 6 months	3940 (3364–4408	3650 (3275–4185)	0.17
Length (cm)	Baseline	52.5 (49.6–54.0)	52.0 (48.5–54.0)	0.36
Δ Length (cm)	Baseline- 3 months	8.6 (6.5–11)	8.5 (6–11.5)	0.81
	Baseline- 6 months	15.5 (13.0–18.7)	15 (12.7–19.0)	0.77
Ponderal Index (kg/m^3^)	Baseline	26.2 (23.8–31.9)	26.4 (24.1–33.0)	0.60
	3 months	26.8 (24.5–30.4)	27.0 (24.8–30.2)	0.67
	6 months	24.5 (23.0–27.1)	24.2 (22.6–26.6)	0.61
Head circumference (cm)	Baseline	36.0 (35.5–37.0)	36.0 (35–36.5)	0.18
Δ Head circumference (cm)	Baseline- 3 months	4 (3.5–5)	4.2 (3.5–4.6)	0.94
	Baseline- 6 months	7.1 (6–8)	7 (6.5–7)	0.64
Less head BMC (g)	Baseline	48 (44–53)	47 (41–51)	0.15
Δ Less head BMC (g)	Baseline- 3 months	−1 ((5)−4)	1.5 ((−5)−5)	0.48
	Baseline- 6 months	10 (4–15)	9 (1–19)	0.62
Less head BMD (g/cm^2^)	Baseline	0.33 (0.30–0.35)	0.31 (0.29–0.35)	0.19
Δ Less head BMD (g/cm^2^)	Baseline- 3 months	0.10 (0.07–0.13)	0.11 (0.08–0.12)	0.36
	Baseline- 6 months	0.12 (0.09–0.15)	0.13 (0.09–0.14)	0.50
FM (g)	Baseline	662 (557–780)	640 (581–737)	0.37
Δ FM (g)	Baseline- 3 months	934 (723–1171)	919 (730–1128	0.65
	Baseline- 6 months	1606 (1264–2002)	1514 (1234–1837)	0.32
LBM (g)	Baseline	3444 (3162–3672)	3277 (3017–3523)	0.10
Δ LBM (g)	Baseline- 3 months	1337 (1101–1727)	1335 (1080–1669)	0.66
	Baseline- 6 months	2469 (2129–2947)	2340 (2094–2748)	0.41

Data presented as median (interquartile range: Q1–Q3). No significant differences between groups, all *P-value>*0.05. (Δ - increment (change) of the study variable between baseline and next visit, *BMD -* bone mineral density, less head BMD - total body less head mineral density, BMC - bone mineral content, less head BMC - total body less head mineral content, LBM - total lean body mass, BMI - body mass index, FM - total fat, android FM – android fat mass, gynoid FM – gynoid fat mass).

## Discussion

During this 6-month intervention study the dynamics of 25(OH)D levels and body composition parameters changes were examined among nursing mothers and their breastfed infants in relation to a vitamin D3 supplementation scheme. In our study maternal median 25(OH)D levels (∼15 ng/ml) at the baseline visit, indicating vitamin D deficiency. More than 60% of mothers and newborns were vitamin D-deficient, and less than 10% of mothers and newborns had 25(OH)D >30 ng/ml. Fortunately, as early as 3 months after implementing vitamin D supplementation, 25(OH)D levels increased. Thereafter, maternal 25(OH)D levels tended to *plateau*, however a slight decline (by ∼1.5 ng/ml) was noted in the 400 IU/d group. Hollis and Wagner [31] reported a much larger decline (by ∼10 ng/ml) of maternal 25(OH)D3 level during the first 3 months of vitamin D supplementation at a dose of 400 IU/d. That difference can be explained, at least in part, by a higher baseline level (∼25–30 ng/ml) and restricted sun exposure in the above-mentioned survey, compared to our population. In our study, women who took 1200 IU/d of vitamin D3 achieved a significantly higher 25(OH)D concentration compared to those supplemented using a dose of 400 IU/d only. However, a vitamin D daily dose of 1200 IU appeared too low to replenish vitamin D deficiency/insufficiency states in all breastfeeding mothers from the study group. It was shown that only 25% of women ingesting 1200 IU/d for 6 months achieved 25(OH) D levels higher than 30 ng/ml, the lowest value of optimal range according to some authors [1], [7], [16], but not IOM experts (25(OH)D >20 ng/ml) [12]. In consequence, fundamental questions of: a) how much vitamin D is enough to reach and maintain optimal 25(OH)D level, and b) what is the optimal range for 25(OH)D levels, remain an issue of debate [32], [33].

There is growing evidence showing serum 25(OH)D levels higher than 30 ng/ml, or even higher than 40 ng/ml as effective for expression of extra-skeletal effects related to proper vitamin D status [1], [34], [35]. On the other hand, the increased target level needs a much higher intake. The studies performed in healthy individuals indicate that vitamin D supplementation at a dose of 3000–7000 IU/d might be necessary to achieve a serum 25(OH)D level of at least 30 ng/ml in 97.5% of that population [17], [35]–[37]. Our study was designed at the beginning of 2009, when recommended supplemental doses of vitamin D were around 400–600 IU/d. Keeping in mind recent literature, to address the question of the lowest effective and safe vitamin D dose needed to observe health benefits for both lactating mother and breastfed offspring, a higher vitamin D doses would be chosen and evaluated in our RCT (1500 IU/d vs. 2000 IU/d vs. 3000 IU/d).

Despite the aforementioned controversies, the improvement in vitamin D status in studied groups was achieved without any evidence of hypervitaminosis D, such as elevated 25(OH)D, hypercalcemia, or increased hypercalciuria. From a clinical point of view, the impact of 1200 IU/d vitamin D dose on the maternal 25(OH)D concentration was very subtle. The final intergroup difference in maternal 25(OH)D concentration was only 2.5 ng/ml. Unfortunately, the maternal compliance at V3 was significantly lower in the 400 IU/d group, which may have influenced the observed 25(OH)D level. When compliant mothers were compared, the difference in 25(OH)D levels between the 400 IU/d and 1200 IU/d groups was not any more significant. However, when the prevalence of vitamin D deficiency in intervention groups was considered, the clinical effect of the higher dose was much more distinct. Vitamin D deficiency affected women in the 400 IU/d group 2 to 3-times more frequently than those in the 1200 IU/d group. Undoubtedly, 400 IU/d of vitamin D appeared unsatisfactory for lactating women. Taking into account the maternal requirement, 1200 IU/d of vitamin D may protect against vitamin D deficiency, but seems too low to fully replenish vitamin D insufficiency (20 ng/ml<25(OH)D <30 ng/ml). Our study findings are in contrast to the last IOM guideline defining the recommended dietary allowance (RDA) for lactating women as 600 IU/d [12]. Maternal supplementation at a dose twice lower than that of our study would likely not result in 25(OH)D >20 ng/ml, at least in a population living at a northern latitude. Though supplementation at a dose of 2000 IU/d was previously reported to increase maternal 25(OH)D to 30–39 ng/ml [35], [38], [39], it still might be insufficient for vitamin D-deficient women [40], [41]. Even higher doses of vitamin D (4000–6400 IU/d) were tested in breastfeeding women as the combined maternal and breastfed infant supplementation [8]–[10], [31], [42]. Those high doses are not universally accepted, although not accompanied by any side effects. It is justified to recommend vitamin D supplementation in breastfeeding women based on their needs and separately in breastfed infants. Our results revealed that maternal vitamin D supplementation at a dose up to 1200 IU/d had no influence on the breastfed offspring’s vitamin D status. Therefore, breastfeeding infants need additional vitamin D supplementation when maternal intake is 1200 IU/d.

Nevertheless, we should keep in mind that each 400 IU/d of vitamin D may increase serum 25(OH)D level by 2.8 ng/ml [17]. We observed a slightly higher maternal 25(OH)D increment during vitamin D supplementation (5.5 ng/ml in the 400 IU/d group and 11 ng/ml in the 1200 IU/d) due to additional input from diet and endogenous skin synthesis [37], [43]. McDonnell’s et al. revealed that every intake of 1 serving per day increases serum 25(OH)D by about 2 ng/ml for eggs and 1 ng/ml for meat and total protein [43]. Interestingly, the fish intake was not associated with serum 25(OH)d level in that study, despite fish being considered a rich source of vitamin D. On the other hand, a mean skin synthesis of vitamin D was 200–650 IU/d at the summer peak, but it accounted for only 10–25% of total basal vitamin D input [37]. In the light of the aforementioned studies, vitamin D supplements seems to be more effective as a source of vitamin D in many contemporary industrialized populations suffering from changing lifestyles (restricted sun exposure, obesity, unhealthy diet).

Another interesting observation relates to PTH as a functional marker of vitamin D status in adults, children and infants but not necessarily in neonates [44]–[47]. We found no correlation between 25(OH)D and iPTH in cord blood, which is in agreement with previous reports [44], [48], [49]. However, a weak correlation between cord blood 25(OH)D level and PTH was also reported [50]. During vitamin D supplementation in our infants, an increasing infants’ 25(OH)D level coincided with an increase in iPTH. However, the inverse association between serum 25(OH)D levels and PTH was noted, as also reported by others [46]. On the other hand, stable and comparable PTH concentrations were reported by Greer in infants supplemented with vitamin D or receiving a placebo, despite significantly lower 25(OH)D levels in the placebo group [45]. Our results indicate that the PTH concentration should not be considered a reliable functional marker of vitamin D status in newborn infants. In contrast, fluctuations in iPTH in breastfeeding mothers during supplementation were in agreement with changes in 25(OH)D. Although a statistically significant negative correlation between maternal 25(OH)D level and iPTH was found, the clinical relevance of such an association is questionable due to its weakness.

We did not find significant differences in any of the body composition variables between intervention groups during supplementation. Keeping in mind the diminutive differences in 25(OH)D levels among the study groups, we assumed that the vitamin D status in nursing women had no impact on bone mass and body composition. On the other hand, a weak negative correlation between maternal 25(OH)D and maternal BMC at the end of the study was noted, but maternal 25(OH)D levels were not associated with maternal total body BMD, lumbar spine BMD, or lumbar spine BMC. The influence of vitamin D status and bone mass in nursing women might be evaluated further but higher vitamin D doses are necessary. The negative impact of maternal fat mass on 25(OH)D level was distinct. The role of android mass should be underlined [51], as the observed increment in maternal 25(OH)D inversely correlated with android fat mass, only. Furthermore, postpartum changes in maternal body composition observed in both intervention groups are in agreement with most previous findings in nursing women [18], [19], [52]. Interestingly, some changes in bone structure geometry also occur, but with minimal impact on bending or compressive strength [52]. The adaptation mechanism during human lactation is still under study. Animal data demonstrate that, instead of vitamin D, calcitriol, or the vitamin D receptor, the increase in intestinal calcium absorption is likely due to the pregnancy-related increase in duodenal expression of the calcium transporter gene *Trpv6* [53]. If the same mechanism exists in humans, increasing the vitamin D intake of lactating women can be presumed to have no beneficial effect on bone mineral content and density. The afore-mentioned speculation is another possible explanation for the lack of intergroup differences in maternal DXA results in our study. Additionally, the dose of maternal vitamin D supplementation had no impact on their infants’ measured DXA variables despite the fact that infants received vitamin D at a dose of 400 IU/d. Gallo et al, also found no differences in BMC between infants supplemented with diverse doses of vitamin D (400–1600 IU/d) [26].

This study has some limitations. First, a small difference was achieved in the 25(OH)D level during supplementation, making it difficult to prove/exclude a positive effect of vitamin D supplementation on bone mass in nursing women. In addition, differences in compliance, maternal education level and final rate of exclusive breastfeeding were observed between study groups, which may have influenced the primary outcome. Finally, dietary vitamin D intake was based solely on fish consumption, not overall food frequency. However, diet is not an important source of vitamin D in Poland [5].

## Conclusions

In summary, our study showed a very high prevalence (∼65%) of postpartum vitamin D deficiency among Caucasian mothers and their newborn offspring living at latitude 52°N. We have demonstrated that vitamin D3 supplementation at a dose of 400 IU/d is not sufficient for nursing women to maintain 25(OH)D >20 ng/ml. Vitamin D3 supplementation at a dose of 1200 IU/d is more effective at improving vitamin D status of nursing women, but still seems too low to fully replenish vitamin D deficiency/insufficiency. Furthermore, maternal vitamin D3 supplementation during lactation at a dose up to 1200 IU/d had no influence on maternal and infant body composition or infant vitamin D status. Further studies of vitamin D supplementation during lactation are necessary to determine the optimal dosing for nursing women when taking into account maternal needs.

## Supporting Information

Checklist S1 **The CONSORT 2010 checklist of information to include when reporting a randomized trial.**
(DOC)

Protocol S1 **The study protocol.**
(DOCX)

Protocol S2 **The study protocol in the original language (Polish).**
(DOC)
