# Supplementary material for: Impact of Vitamin D Supplementation during Lactation on Vitamin D Status and Body Composition of Mother-Infant Pairs: A MAVID Randomized Controlled Trial
Source: PLoS One. 2014 Sep 18;9(9):e107708. doi: 10.1371/journal.pone.0107708 (PMC4169453; doi:10.1371/journal.pone.0107708)
Supplement: Protocol S1 — The study protocol. (DOCX) [file pone.0107708.s002.docx]

Protocol S1. The study protocol.

**The study title**: ”Influence of vitamin D supplementation during lactation on vitamin D status, bone mass and body composition in lactating mothers and their breastfed infants”

**Introduction**

Lactating women and their offspring are at risk of vitamin D deficiency. Vitamin D supplementation at a dose of 400 IU/d is not sufficient for lactating women, and higher doses are required (up to 2000 IU/d). Vitamin D supplementation at a dose of 4000-6000 IU/d during lactation was safe for both lactating women and their offspring. However, the safety of such high doses has only been proved in studies with a small number of participants. The safety and effectiveness of vitamin D supplementation at doses higher than 400 IU/d have not yet been studied in Polish lactating women. A high incidence of vitamin D deficiency in pregnant and lactating women and their offspring has been reported. An appropriate vitamin D intake is a fundamental issue because of the role of vitamin D, not only in calcium and bone homeostasis, but also in many other physiological functions. Vitamin D may have an influence on the musculoskeletal system (body composition parameters and interactions between them).

**Aim of the study**

We aim to prospectively compare the influence of vitamin D supplementation (400 IU/d vs. 1200 IU/d) on the vitamin D status, bone mass and body composition of lactating women and their offspring, taking safety issues into account. Additionally, we want to prove that vitamin D supplementation at a dose of 400 IU/d is sufficient to build up an appropriate vitamin D status in breastfed infants, irrespective of maternal vitamin D supplementation (up to 1200 IU/d). We also aim to assess the influence of the season of delivery on the maternal and newborn’s vitamin D status.

**Patients**

150 lactating mother-infant pairs.

*Inclusion criteria*

Healthy women, declaring breastfeeding for 6 months.

Breastfed term newborns without major medical problems.

*Exclusion criteria*

Maternal diabetes, maternal endocrine disorders during pregnancy, multiple gestation.

Liver diseases and cholestasis, renal insufficiency, endocrine disorders, congenital malformations and anticonvulsant treatment in neonates.

**Methods**

This is a prospective double-blind randomized control trial. Intervention in mothers will last for 6 months. Lactating mothers will be randomly allocated to two groups with different vitamin D intakes.

Group 1 – Maternal vitamin D supplementation at a dose of 400 IU/d (multivitamin tablet containing 400 IU of cholecalciferol + placebo),

Grupa 2 – Maternal vitamin D supplementation at a dose of 1200 IU/d (multivitamin tablet containing 400 IU of cholecalciferol + 800 IU of cholecalciferol),

All infants will receive 400 IU/d of vitamin D3 (1 capsule or 1 drop) throughout breastfeeding, according to the Polish recommendation.

The following assessments are planned during the study:

- Maternal and infants’ data (gestational age, birth weight, Apgar score, vitamin D intake from diet and supplements, educational/socioeconomic status, duration of sunlight exposure, usage of sun blockers)
- Anthropometric measurements (weight, height/length, maternal BMI)
- Assessment of vitamin D status (measurement of serum 25 hydroxyvitamin D (25-OHD) and serum parathormone (PTH))
- Assessment of biochemical signs of vitamin D overload (serum calcium, urinary calcium and creatinine (urinary calcium /creatinine ratio)).
- Assessment of bone status (total body and lumbar spine bone mineral content (TB BMC L1-L4 BMC), total body and lumbar spine bone mineral density (TB BMD, L1-L4 BMD) by dual energy X-ray absorptiometry
- Assessment of body composition (lean body mass (LBM) and fat mass (FM)) by dual energy X-ray absorptiometry
- Statistical analysis (distribution analysis, Student’s T test for normally and U-Mann-Whitney test for non-normally distributed variables, chi^2^ test for categorical variables, Pearson or Spearman correlation, one-way analysis of variance or Friedman ANOVA test). *P*-values <0.05 were considered significant.

*Schedule of study visit*

Baseline assessment (before introduction of vitamin D supplementation) will provide baseline characteristics of the studied participants. Cord blood samples will be collected at delivery. Maternal baseline blood samples will be collected within 3 weeks after delivery when maternal and infants’ DXA measurements will be performed. Follow–up visits will take place at the 3^rd^ and 6^th^ month after delivery.

Biochemical measurements in serum will be performed by fully automatic methods to minimize collected blood volume at 2ml.

A single dual energy X-ray absorptiometry measurement will be performed at every visit for assessment of bone status and body composition, to minimize the radiation dose at 5 μSv (for comparison, the radiation dose for spinal X-ray is 500-900 μSv, and for a transatlantic flight, 60 μSv)

**Theoretical results**

- Confirmation of the hypothesis that maternal vitamin D supplementation at a dose of 1200 IU/d is more efficient than 400 IU/d for lactating women, and safe for both mother and child, and positively changes bone mass and body composition (better ratio between muscle and fat components)
- Verification of a maternal need for vitamin D supplementation during lactation and modification of current recommendations
- Confirmation of the effectiveness of vitamin D supplementation at the currently recommended dose in breastfed infants
- Confirmation of the hypothesis that the season of delivery has an influence on maternal and newborn vitamin D status, and if so, a change in the currently recommended vitamin D supplementation according to season.

**Benefits for patients**

This study will help to optimize prophylactic strategies against vitamin D deficiency in lactating women and their offspring, and also protect against the long-term medical consequences of vitamin D deficiency. It is well-known that vitamin D deficiency increases the risk of osteoporosis, autoimmunological diseases, cancers and cardio-vascular diseases. This study may improve the quality of women’s and children’s lives in the future.
