# Supplementary material for: Impact of Vitamin D Supplementation during Lactation on Vitamin D Status and Body Composition of Mother-Infant Pairs: A MAVID Randomized Controlled Trial
Source: PLoS One. 2014 Sep 18;9(9):e107708. doi: 10.1371/journal.pone.0107708 (PMC4169453; doi:10.1371/journal.pone.0107708)
Supplement: Protocol S2 — The study protocol in the original language (Polish). (DOC) [file pone.0107708.s003.doc]

Protocol S2. The study protocol in the original language (Polish).

PROTOKÓŁ BADANIA

**„**“Wpływ suplementacji witaminą D w okresie laktacji na zasoby ustrojowe witaminy D, masę kostną oraz skład ciała matek karmiących oraz ich dzieci karmionych piersią.”

**Wstęp**

Matki karmiące oraz ich potomstwo są w grupie ryzyka niedoborów witaminy D. Dotychczas zalecana podaż witaminy D w dawce 400 IU/d z preparatu wielowitaminowego nie pokrywa potrzeb matki karmiącej. Wyniki badań wskazują, iż dla utrzymania optymalnych zasobów witaminy D u matki konieczne jest stosowanie zdecydowanie wyższych dawek (do 2000 IU/d). Wykazano, że nawet dawki 4000-6000 IU/d były bezpieczne dla matki i dziecka aczkolwiek były to badania na małych grupach. Brak jest danych o efektywności i bezpieczeństwie suplementacji witaminą D w wyższych dawkach (>400 IU/d) w populacji polskich kobiet karmiących piersią. Polskie badania wskazują, ze niedobór witaminy D dotyczy znacznego odsetka kobiet ciężarnych i ich potomstwa po urodzeniu, dlatego można się spodziewać, że problem ten dotyczy również okresu laktacji. Zapewnienie optymalnej podaży witaminy D nabiera szczególnego znaczenia w kontekście jej plejotropowego działania i wpływu nie tylko na układ szkieletowo-mięśniowy (skład ciała i wzajemne relacje między tkankowymi komponentami ciała), ale również wiele innych narządów i funkcji fizjologicznych.

**Cel pracy**

Celem pracy jest prospektywna analiza porównawcza zasobów witaminy D i ich wpływu na masę kostną i skład ciała matek karmiących i ich potomstwa w zależności od poziomu suplementacji witaminą D w okresie laktacji (400 IU/d vs. 1200 IU/d) z oceną bezpieczeństwa u matki i dziecka. Dodatkowo chcemy potwierdzić, że suplementacja niemowlęcia karmionego piersią w dawce 400 IU/d jest odpowiednia dla zbudowania prawidłowych zasobów witaminy D niezależnie od suplementacji matki karmiącej w dawce do 1200 IU/d. Celem pracy jest również ocena wpływu pory roku na zasoby ustrojowe witaminy D u matki i dziecka w momencie porodu

**Pacjenci**

*150 par matka karmiąca piersią –dziecko*

*Kryteria włączenia*

Zdrowe kobiety, deklarujące wyłączne karmienie piersią przez 6 miesięcy

Noworodki donoszone, bez istotnych problemów zdrowotnych karmione piersią

*Kryteria wykluczenia*

Cukrzyca, zaburzenia endokrynologiczne w ciąży, ciąża mnoga

Choroby wątroby i dróg żółciowych, niewydolność nerek, zaburzenia endokrynologiczne, zespoły wad wrodzonych, leki p/drgawkowe u noworodka.

**Metodyka badań**

Jest to prospektywne badanie z randomizacją z podwójnie ślepą próbą. Okres interwencji wynosi 6 miesięcy. Losowa alokacja matek karmiących piersią do dwóch równolicznych grupa badanych o różnym poziomie suplementacji witaminą D w okresie laktacji:

*Grupa 1 –*matki suplementowane witaminą D3 w dawce 400 IU/d (preparat wielowitaminowy + placebo),

*Grupa 2 –* matki suplementowane witaminą D3 w dawce 1200 IU/d (400 IU/d- preparat wielowitaminowy + 800 IU/d cholecalciferol),

Niemowlęta będą otrzymywać witaminę D3 w dawce dziecko 400 IU/d zgodnie z aktualnymi polskimi rekomendacjami (standardowe postępowanie)

Planowana jest:

- Analiza danych z wywiadu (wiek płodowy, masa urodzeniowa ciała, punktacja Apgar, podaż witaminy D_3_ z diety i preparatów farmakologicznych, status socjoekonomiczny, czas ekspozycji na słońce, stosowanie filtrów UV)
- Pomiary antropometryczne (masa ciała, wysokość/ długość), BMI u matek
- Ocena zaopatrzenia organizmu w witaminę D (oznaczenie parametrów 25(OH)D i PTH w surowicy krwi)
- Ocena objawów biochemicznych przedwakowania witaminy D (Ca w surowicy, wydalanie Ca z moczem ( wskaźnik Ca/kreatynina w porcji moczu).
- Densytometryczna ocena statusu kostnego (oznaczenia parametrów TBBMC i L1-L4BMC oraz TBBMD i L1-L4BMD)
- Densytometryczna ocena składu ciała (ozaczenia parametrów LBM oraz FM)
- Analiza statystyczna danych (analiza rozkładu, test T studenta dla zmiennych powiązanych i niepowiązanych, test U-Manna-Whitneya, jedoczynnikowa analiza wariancji oraz test Friedmana, korelacja Pearsona/Spermna, test chi^2^ . Poziom istotności statystycznej przyjęto dla p < 0,05.

*Harmonogram wizyt*

Wyjściowe oznaczenia zostaną wykonane przed rozpoczęciem suplementacji witaminą D. Po porodzie zostanie zabezpieczona krew pępowinowa a badanie densytometryczne u dziecka i matki oraz pobranie krwi od matki zostanie wykonane w ciągu pierwszych 3 tyg po porodzie). Badania kontrolne planowane są po 3 i 6 miesiącach po porodzie.

Badania biochemiczne w surowicy krwi – badania wykonywane będą w surowicy metodą w pełni automatyczną, co pozwala ograniczyć objętość pobieranej krwi do 2ml

Badania densytometryczne – wszystkie parametry densytometryczne mierzone będą podczas jednego badania, gdzie dawka promieniowania jonizującego wyniesie nie więcej niż 5 μSv (dla porównania: rtg kręgosłupa = 500-900 μSv, przelot transatlantycki = 60 μSv)

**Oczekiwane wyniki**

- weryfikacja hipotezy o wyższej skuteczności i bezpieczeństwie suplementacji witaminą D w dawce 1200 IU/d w porównaniu do 400 IU u matki karmiącej na jej zasoby witaminy D oraz potencjalnym korzystnym wpływie na masę kostną i skład ciała (lepsze proporcje kompartmentu mięsniowego do tłuszczowego) u matki i dziecka
- weryfikacja postępowania normalizującego zaopatrzenie w witaminę D u matek karmiacych piersią z ew. modyfikacja dotychczasowych zaleceń
- potwierdzenie skuteczności suplementacji witaminą D w dotychczas zaleceanej dawce u niemowląt karmionych piersią
- weryfikacja hipotezy o wpływie pory roku na zasoby witaminy D u kobiety ciężarnej i nowrodka z ew. modyfikacją zalecanej podaży witaminy D w zalezności od pory roku

**Korzyści dla pacjenta**

Badanie to przyczyni się do optymalizacji postępowania w zakresie profilaktyki niedoborów witaminy D u kobiet w okresie laktacji oraz u ich potomstwa. Ma ono na celu zapobieganie konsekwencjom niedoborów witaminy D również w dłuższej perspektywie czasowej. Wiadomo bowiem, że niedobory witaminy D to zwiększone ryzyko osteoporozy, chorób o podłożu autoimmunologicznym, nowotworów oraz chorób sercowo- naczyniowych. Badanie to przyczyni się więc do poprawy w przyszłości jakości życia kobiet i ich dzieci.
